# A software tool for the quantification of metastatic colony growth dynamics and size distributions *in vitro* and *in vivo*

**DOI:** 10.1371/journal.pone.0209591

**Published:** 2018-12-27

**Authors:** Soumitra Bhoyar, Inês Godet, Josh W. DiGiacomo, Daniele M. Gilkes

**Affiliations:** 1 Department of Chemical and Biomolecular Engineering, The Johns Hopkins University, Baltimore, Maryland, United States of America; 2 Department of Oncology, The Sidney Kimmel Comprehensive Cancer Center, The Johns Hopkins University School of Medicine, Baltimore, Maryland, United States of America; Pennsylvania State Hershey College of Medicine, UNITED STATES

## Abstract

The majority of cancer-related deaths are due to metastasis, hence improved methods to biologically and computationally model metastasis are required. Computational models rely on robust data that is machine-readable. The current methods used to model metastasis in mice involve generating primary tumors by injecting human cells into immune-compromised mice, or by examining genetically engineered mice that are pre-disposed to tumor development and that eventually metastasize. The degree of metastasis can be measured using flow cytometry, bioluminescence imaging, quantitative PCR, and/or by manually counting individual lesions from metastatic tissue sections. The aforementioned methods are time-consuming and do not provide information on size distribution or spatial localization of individual metastatic lesions. In this work, we describe and provide a MATLAB script for an image-processing based method designed to obtain quantitative data from tissue sections comprised of multiple subpopulations of disseminated cells localized at metastatic sites *in vivo*. We further show that this method can be easily adapted for high throughput imaging of live or fixed cells *in vitro* under a multitude of conditions in order to assess clonal fitness and evolution. The inherent variation in mouse studies, increasing complexity in experimental design which incorporate fate-mapping of individual cells, result in the need for a large cohort of mice to generate a robust dataset. High-throughput imaging techniques such as the one that we describe will enhance the data that can be used as input for the development of computational models aimed at modeling the metastatic process.

## Introduction

Metastasis is the migration of cancer cells from the primary tumor to distant organs to form secondary tumors. It involves a series of steps, including detachment from the primary tumor, intravasation, extravasation at a secondary site, invasion and proliferation [1,2]. The majority of cancer-related deaths are due to metastasis [3–5], and experimental mouse models are required to study metastasis. In order to study the metastatic process in such experimental models, the ability to quantify the extent of the metastases is needed. However, current methods of quantitatively analyzing metastases *in vivo* do not provide sufficient quantitative information pertaining to metastatic colonies or their spatial distributions at the organ-level [6].

Different methods exist for analyzing the extent of experimental metastasis. Traditionally, Hematoxylin & Eosin (H&E) stained tissue sections have been used to visualize metastatic regions in organs. Region-Of-Interest (ROI) tracing on sections can provide an estimate of individual metastatic colony sizes. However, this is highly time-consuming, especially when dealing with organs that have a large metastatic burden and a large number of samples and images. It is also difficult for the experimenters to differentiate between micrometastases and immune infiltration in lung tissue [7]. Work on automated segmentation by Image analysis with H&E staining is in progress, but remains problematic especially with micrometastatic nodules (containing 4–5 cells) [8]. Another tool used to quantify overall metastatic burden in mice organs is the quantitative polymerase chain reaction (qPCR). However, since qPCR is performed on DNA extracts of organ tissues, the spatial fidelity of metastatic colonies is not maintained. Hence no information of the size of metastatic colonies or their distribution can be obtained [6,9]. Bioluminescence imaging (BLI) is a popular method of quantifying tumor and metastatic loads based on measuring fluorescence values from cancer cells engineered to emit photons via the luciferase expression. Tumor cells are genetically modified to express firefly luciferase and introduced in the animal, where they form tumors *in vivo*. Upon injection of the substrate luciferin, the tumor cells emit a signal that can be measured with a bioluminescence imager, while the mouse is anesthetized [10]. However, BLI suffers from rapid signal attenuation in tissues hence any signal received is significantly weighted toward cells localized closer to the surface of the organ. Furthermore, the method is affected by significant mouse-to-mouse variation since the light-emitting reaction depends on a variety of factors (depth, concentrations of luciferin, ATP and oxygen) [11].

Digital pathology has gained increasing importance over the past decade with increases in computational power and the advent of sophisticated image analysis techniques. While traditional visual microscopy based pathology is the current gold-standard, it can be prone to lack of standardization or variation between pathologists [12,13], and can be time-consuming [14]. These are the principal issues which digital pathology seeks to overcome. While digital pathology is slowly gaining relevance in the clinical setting, there are some technical issues which remain to be tackled [15–17]. For example, three-dimensional cell clusters or dense smears make digital pathology difficult [18]. In research applications involving fluorescently labeled cells, some of the limitations encountered in the clinical setting do not apply. Fluorescently labeled cells are a popular choice *in vitro* and are becoming increasingly widespread for *in vivo* experiments [19–21]. From a digital point-of-view, fluorescent cells have an advantage in that they are easier to identify and can be segmented into colonies with greater ease than clinical specimens stained with colorimetric dyes such as H&E. This facilitates the application of image analysis techniques in obtaining quantitative data from histological sections.

This paper presents a high-throughput image analysis scheme to obtain size distributions of lung metastases, including the ability to estimate number of cells within a metastatic colony, colony size and colony counts for fluorescently labeled cells. This method is sensitive enough to identify micrometastases consisting of 4–5 cells. Obtaining quantitative metrics would be particularly useful in investigating clonal competition in genetically heterogenous tumors and visualizing genetic exchange between cancer cells both *in vivo* and *in vitro* [22–24]. Furthermore this method would be useful for computational modeling of cancer metastasis. One of the motivations for a predictive model for metastasis is to obtain metastatic colony size distributions and hence estimate the formation of micrometastases that are below the detection limit of clinical diagnostics [25,26]. As of now, there are a limited number of mathematical models that describe metastasis as opposed to models describing tumor growth, which usually use BLI or computational tomography for their experimental validation [27]. The lower detection limit, higher resolution, consistency and speed of the method presented here would assist in obtaining quantitative data for the purpose of developing and experimentally validating mathematical models of metastasis [28–30].

Apart from *in vivo* analyses, this methodology can be applied to *in vitro* systems as well. Clonogenic assays are a widely-used tool for cancer biologists and are used to obtain the growth potential of cells and to quantify differences in sensitivity to cytotoxic agents [31]. However, clonogenic assays are not suitable for co-culturing different cell lines and are unable to visualize proteins of interest, and unable to accurately segment individual cells or colonies when they are close together. The assay described in this paper provides confluence, colony cell-count and size distributions, and reveals synergistic and co-culture effects in fluorescently labeled cells. Furthermore, the tool is able to quantify expression of immunofluorescently labeled proteins at the colony level. To demonstrate this, we used the proliferation marker Ki-67 as our protein of interest. The methods described here are especially relevant for lineage tracing in populations of cells labeled with a reporter construct [32,33].

## Materials and methods

### Cell culture

MCF7 and MDA-MB-231 cells were cultured in Dulbecco's Minimum Essential Media (DMEM) (Sigma-Aldrich, St. Louis MI—USA) supplemented with 10% Fetal Bovine Serum (FBS) and 1% penicillin-streptomycin (Invitrogen, Carlsbad) in a humidified atmosphere of 5% CO_2_ at 37°C.

### Fluorescent cell lines

DsRed-expressing MDA-MB-231 and MCF-7 cell lines were generated by transducing them with vector 1 as shown in S1A Fig. Vector 1 expresses a DsRed reporter with a stop codon flanked by tandem loxP sites (‘floxed’) located adjacent to a GFP gene. To generate GFP-expressing cells, we transduced the DsRed-expressing cell lines with Cre-recombinase (vector 2, S1B Fig). Deletion of the DsRed site by Cre-recombinase results in the rapid loss of DsRed protein and permanent GFP expression. All cell lines were sorted using a SH800 Cell Sorter in sterile conditions, to guarantee purity and high fluorescence levels.

### *In vitro* colony formation assay

The methods described in this section were used as a part of the *in vitro* colony analysis scheme.

#### Preculture in Paclitaxel

Prior to plating, GFP-expressing cells were cultured in media containing 2 nM Paclitaxel for 48 hours. DsRed-expressing cells were cultured in normal media alone. After 48 hours of preculture, 2000 DsRed+ and 2000 GFP+ cells were mixed and co-cultured in a 6-well plate.

#### Fixing and staining

Cells were fixed by removing culture media, adding 4% paraformaldehyde (PFA) in phosphate-buffered-saline (PBS) for 15 minutes and washing with PBS. Next, to stain for Ki-67, 1% Triton-X was added for 5 minutes, followed by 2% bovine serum albumin (BSA) for 30 minutes, and finally a 1:100 solution of anti-Ki-67 monoclonal antibody (Alexa Fluor 647 Mouse anti-Ki-67, BD Biosciences) in BSA for 90 minutes. The wells were washed with PBS after each step. After Ki-67 staining, nuclei were stained by adding 5 μg/mL Hoechst 33342 solution in PBS for 15 minutes. Fixed and stained samples were stored (at 4°C) in PBS.

#### Imaging well plates

Well plates were imaged using a Cytation 5 imaging reader (BioTek, Winooski, VT, USA) equipped with an incubated stage, which was set at 37°C when imaging live cells. Plates were imaged at a magnification of 4x. DAPI, GFP, RFP and CY5 images were obtained when imaging fixed and stained cells, whereas only GFP and RFP channels were used for live cell imaging. A montage of 180-images in each channel was obtained such that it covered approximately 80% of the well area, which provided a sufficient number of colonies to obtain size distributions. The images were stitched with NIS Elements software (Nikon Instruments Inc., Melville, NY, USA) and reduced by 50% to facilitate processing.

### Mice

Female 5- to 7-week-old NSG mice were used according to protocols approved by the Johns Hopkins University Animal Care and Use Committee. Mice were anesthetized, and 5 × 10^4^ MDA-MB-231 cells were directly injected into the tail vein, in a mixture of 1:1 DsRed- and GFP-expressing cells. After 3 weeks, the mice were sacrificed and the lungs were inflated with a solution of PBS and OCT(Optimal Cutting Temperature Compound, Fisher Healthcare, Pittsburgh, PA, USA), harvested and immediately fixed in 10% neutral buffered formalin for 1 hour, followed by an overnight soak in 30% sucrose solution at 4°C. The lungs were then frozen in OCT and cryosectioned into 15 μm slices, that were immediately stored at -20°C.

### *In vivo* metastatic colony analysis

The methods described in this section were used as a part of the *in vivo* scheme to analyze metastases in lung sections.

#### Preparing tissue slides

Tissue slides were washed 3 times in a PBS-T solution (1% Tween in PBS 1X) and incubated with Sudan Black (0.1% w/v) for 25 minutes at room temperature to reduce tissue background fluorescence. After 3 additional PBS-T washes the tissue slides were stained with DAPI (1:1000 in PBS) for 15 minutes at room temperature. After washing, the slides were mounted with a 90% Glycerol mounting solution and covered with a coverslip that was sealed with nail polish.

#### Imaging tissue sections

Similar to the in vitro imaging step, slides were imaged at a magnification of 4x in the DAPI, GFP and RFP channels. A montage of images was used to cover the entire lung section on the slide, and the images produced were stitched using Gen5 (BioTek). The stitched images were reduced to 50% in size in order to facilitate processing.

#### Statistical analysis

Differences in median colony sizes were verified through a Wilcoxon Rank Sum test, and distributions were analyzed by the Kruskal-Wallis test. Ki-67 expression was analyzed using an unpaired t-test. All tests were evaluated through functions available in MATLAB (Mathworks, Natick, MA, USA) or GraphPad Prism (GraphPad Software, Inc., San Diego, CA, USA).

## Results

### *In vitro* colony analysis of fixed samples

**Fig 1:**
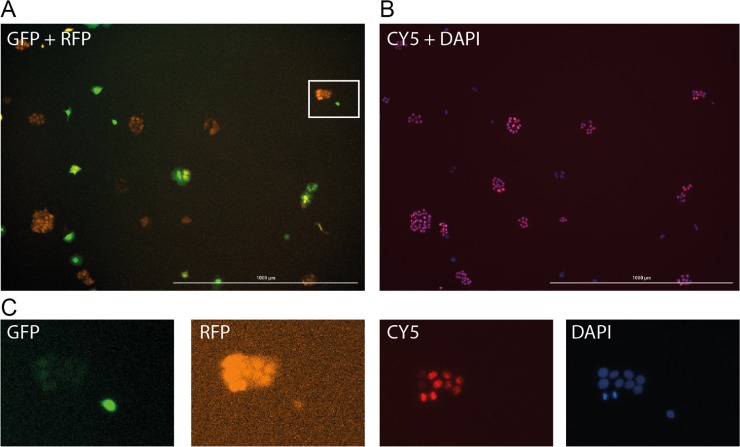
Fixed and stained *in vitro* images. A: GFP, RFP channel overlay. B: DAPI, CY5 (Ki-67) channel overlay. C: Individual channels from the inset region shown in 1A. Scalebar = 1 mm.

**Fig 2:**
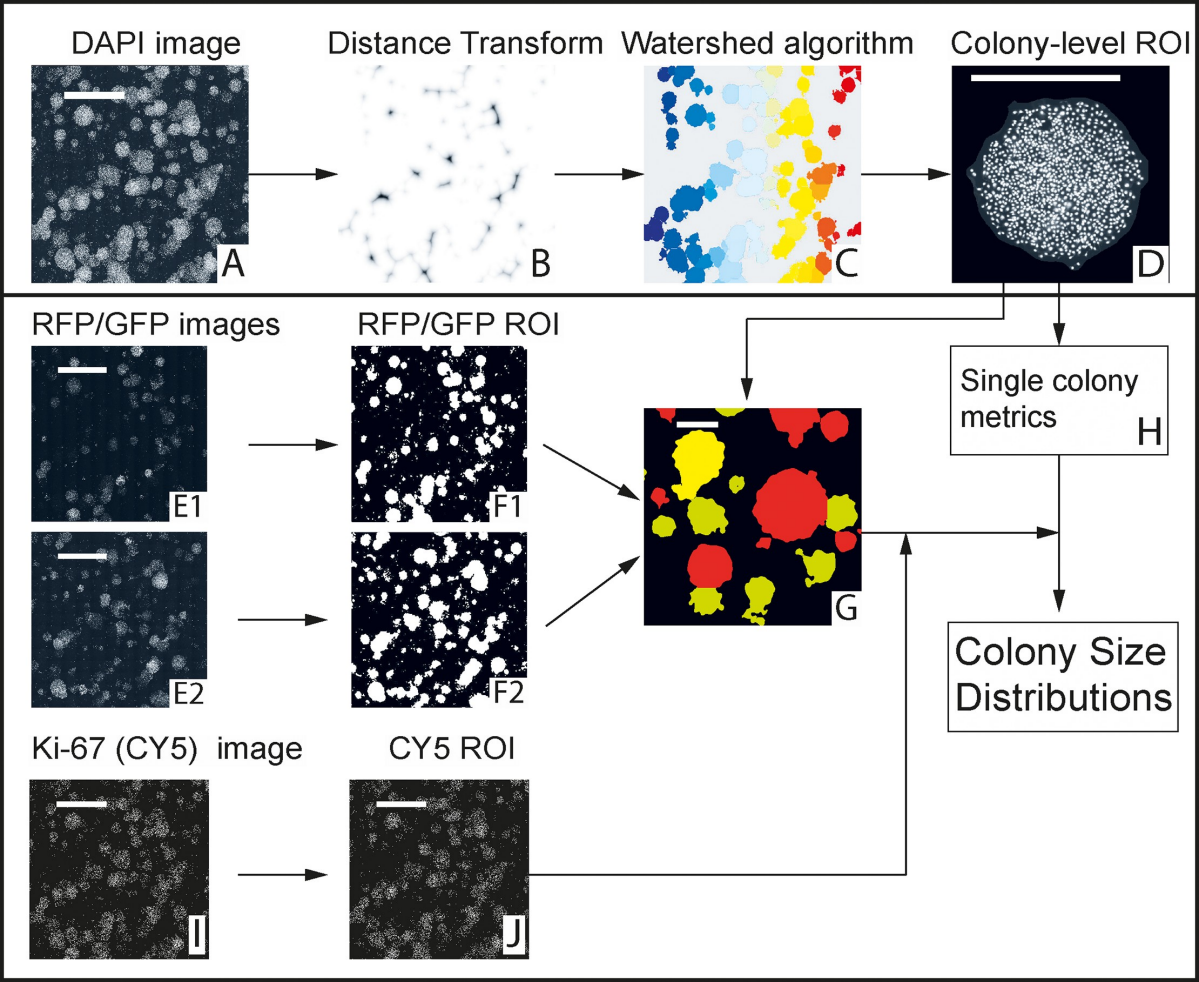
Image processing steps for *in vitro* images. A: Nuclear staining (DAPI channel). B: Preprocessing steps and distance transform identifies watersheds. C: Segmentation of colonies by watershed algorithm. D: Individual colonies analyzed. E: DsRed and GFP signal captured in RFP and GFP channels respectively. F: DsRed+/GFP+ regions obtained by preprocessing steps and binarization. G: Segmented colonies classified as DsRed+/ GFP+ based on input from images F1 and F2. G: Cell count and area of individual colonies obtained from colony-level DAPI image. I: Ki-67 staining imaged in the CY5 channel. J: Regions of MCF7 cells expressing Ki-67 obtained by binarization. Scale bars: 5 mm (A, E, I, J), 1 mm (G, D).

We developed a ready-to-use MATLAB script (available from GitHub: https://github.com/sbhoyar1/ColonySizeDistributions) to determine colony size distributions, proliferation and to analyze protein expression at the colony level. To demonstrate the utility of the algorithm, we prepared fixed and stained colonies of GFP-expressing (GFP+) and DsRed-expressing (DsRed+) MCF7 cells. We introduced differences in growth rates and colony sizes between the GFP+ and DsRed+ cell lines by preculturing GFP+ cells in 2 nM Paclitaxel for 48 hours. DsRed+ cells were pre-cultured in drug-free media. Next, we mixed 2000 of each GFP+ and DsRed+ cells and plated them in 6-well plates. On day 2 and 4, cells were fixed, stained and imaged. Nuclei were stained with DAPI and the proliferation marker, Ki-67 (CY5). Images were taken in the DAPI, CY5, GFP and RFP channels. We observed that the GFP+ cells (which were pre-treated with Paclitaxel) formed smaller colonies with a lower proportion of Ki-67 expressing cells (Fig 1). Each image was analyzed using the following image-processing scheme. The image processing steps involved in segmenting fixed and immunostained colonies *in vitro* are given in Fig 2 and described below.

#### Preprocessing

The following preprocessing steps were applied to each image (Fig 2A, 2E1, 2E2 and 2I) to reduce background signal and lens artifacts:

1. Top-hat filtering is applied to the image as a background correction step.
2. Gaussian filtering is utilized to eliminate jagged boundaries and obtain smooth colony-regions.
3. A final contrast adjustment step ensures that the fluorescent regions are above the segmentation threshold.

The results of the preprocessing steps are shown in S2A and S2B Fig. After this step, the image is binarized. The binarized image consists of pixels of size 1.81 um^2^ labeled with ‘1’ if they are part of a colony and ‘0’ if they are part of the background.

#### Distance transform

A distance transform is then performed on the complement of the binarized image (Fig 2B), which effectively replaces the value of each pixel within a colony with the Euclidean distance to the nearest boundary. The image shown is inverted, and the intensity of the background pixels was raised to saturation (S2C and S2D Fig) The dark regions at the center of each colony function as catchment regions for a watershed segmentation step. This method takes advantage of the fact that the colonies are roughly circular. The top-hat filter applied in the preprocessing stage mitigates uneven illumination, which otherwise leads to a variation in greyscale intensity values and affects segmentation accuracy. This, and the smoothening effect of the Gaussian blur is sufficient to eliminate noise, which eliminates the need for a median filtering step, and ensures that the subsequent watershed segmentation step successfully identifies colony regions. Finally, we use an h-minima transform to suppress small minima which would otherwise lead to over-segmentation.

#### Watershed segmentation

The watershed transform is a commonly used method for image segmentation. The algorithm views the subject image as a topographical map, viewing bright pixels as `high' and dark ones as `low'. It segments the image into catchment areas based on dark 'basins', which are then used as foci for segmenting objects corresponding to each basin [34,35]. In our application, we use a distance transform to obtain 'high' or bright regions, invert them so that they become 'basins', and subsequently use them as foci for segmenting colonies *in vitro* (Fig 2C). An example of the segmentation step is provided in S3 Fig, where a large group of connected colonies is accurately segmented into individual colonies. While the script works well with clustered or partially overlapping colonies, it cannot distinguish sheet-like areas or areas where multiple colonies may have fused into a `super-colony'.

#### Colony-level analysis

The output of the segmentation step is a complete colony map that contains the size (in terms of both area and number of constituent cells) and location of each colony of cells in a given well (Fig 2D and 2H). The number of constituent cells is obtained by counting nuclei in the DAPI channel image. The algorithm for segmenting nuclei closely resembles the colony segmentation step in that it also involves a distance transform followed by an application of the watershed algorithm. Segmented colonies are then classified as DsRed+ or GFP+ based on input from the binarized RFP/GFP channel images (Fig 2E–2G). This is sufficient information to obtain distinct colony size distributions of the two cell lines (DsRed+ and GFP+) in co-culture and make quantitative comparisons.

#### Ki-67 analysis

**Fig 3:**
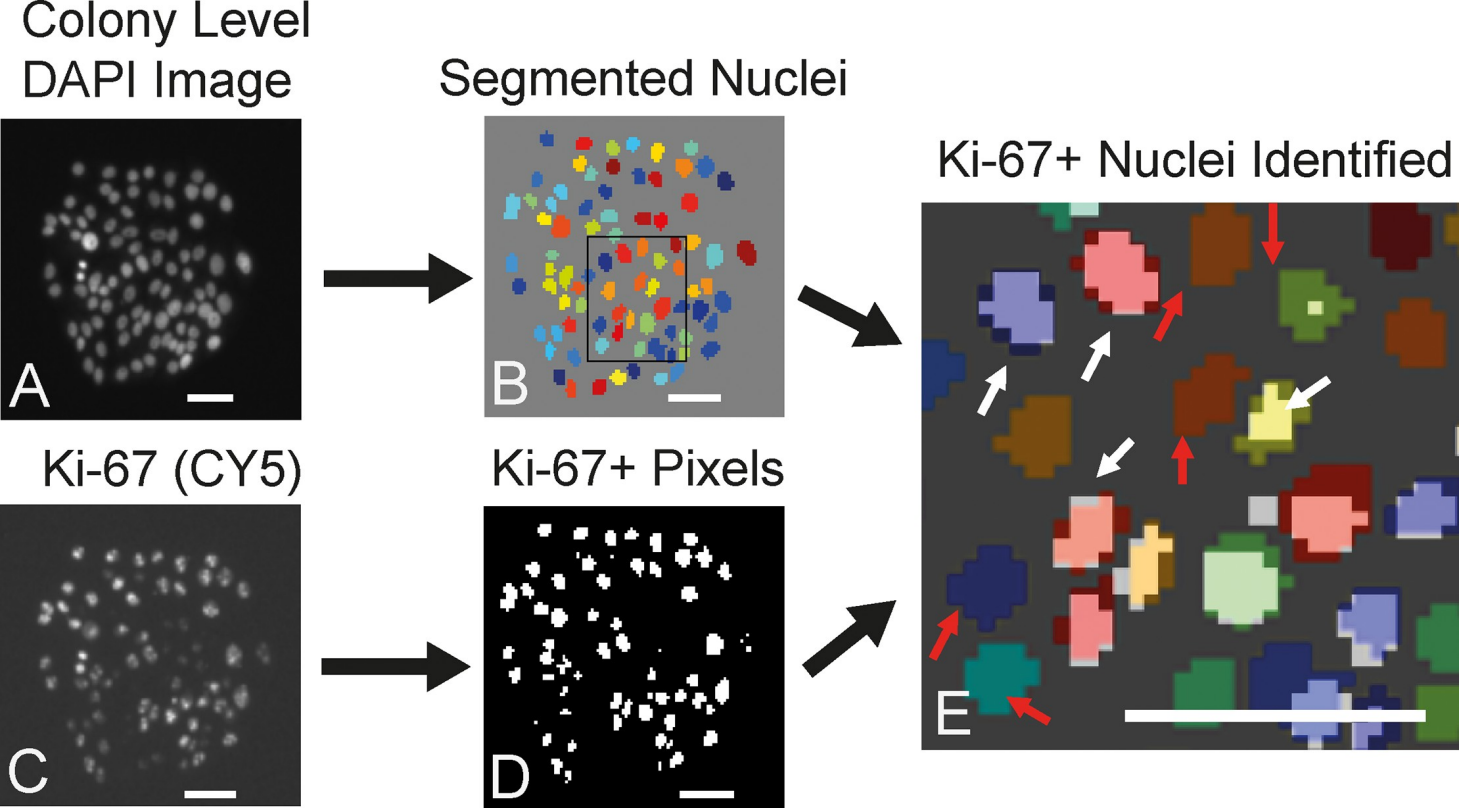
Ki-67 analysis. **A: DAPI Image.** B: Segmented Nuclei. C: Ki-67 staining image (CY5 Channel). D: Binarized Ki-67 Stain. E: Overlap of B and D, to identify Ki-67+ nuclei (white arrows) and Ki-67- nuclei (red arrows). Scale bars = 50 microns.

Using our script, we quantify expression of the proliferation marker Ki-67 within each colony (Fig 2I, 2J and Fig 3). In order to do this, nuclei are segmented in the DAPI channel using a distance-transform followed by application of the watershed algorithm, similar to the scheme described previously for segmenting whole colonies (Fig 3A and 3B). Ki-67 was stained using a conjugated antibody that was imaged in the CY5 channel (Fig 3C). The pixels constituting each nucleus (in the DAPI channel) are compared with corresponding pixels in the binarized Ki-67 image (Fig 3D). Thus the fraction of total pixels (or area) positive for Ki-67 are obtained for each nucleus, i.e. the fraction of nuclear area that overlaps with the Ki-67 signal. If this fraction is greater than a fixed arbitrary threshold (which is 0.25 in our assay), the nucleus is considered to be positive for Ki-67. Thus, we can identify and count the cells that express ki-67 in each colony (Fig 3E). The distribution of this metric can be obtained for the entire population of colonies and compared. While we have limited our analysis to Ki-67, this assay can be applied to any protein of interest that can be stained and imaged using immunofluorescent labeling.

#### *In vitro* fixed-cell experimental results: Paclitaxel pretreatment reduces median colony size and Ki-67 expression levels

**Fig 4:**
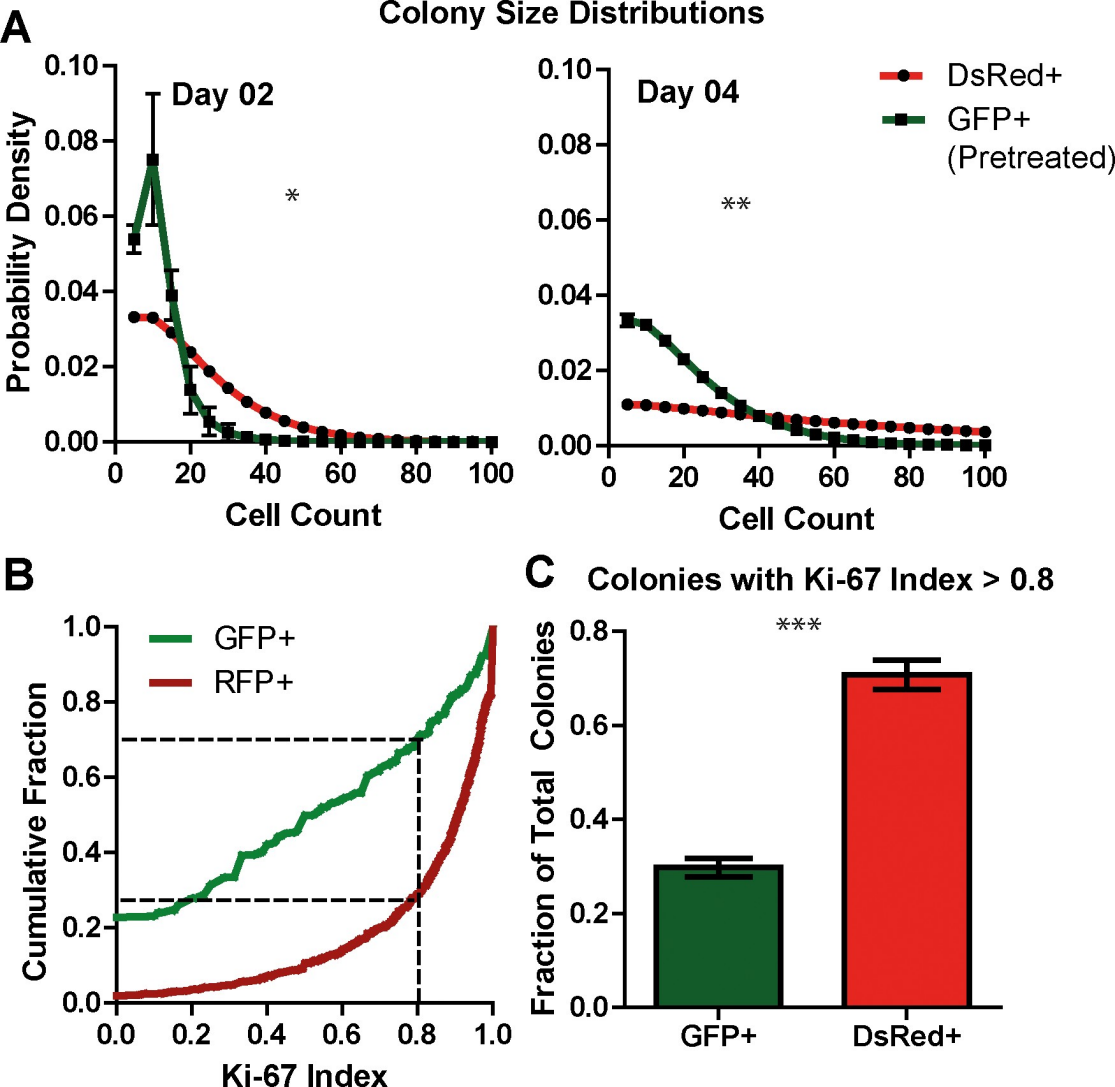
*In vitro* results. A: Colony size distributions 2 days and 4 days after plating. The Y-axis shows probability density (relative probability) and the X-axis shows colony size in number of cells. Mean and standard deviations are shown for populations of colonies (n >150, per well) obtained from a set of 3 wells of a 6 well-plate and represent biological replicates. Significance was determined using the two-tailed Wilcoxon rank-sum test. B: Cumulative frequency distribution of Ki-67 index over a population of colonies (n >150, per well) obtained from a set of 3 wells of a 6 well-plate. Dashed lines show Ki-67 index value of 0.8. C: Fraction of cells with Ki-67 index > 0.8, showing mean and standard error over 3 wells. Significance was determined using unpaired t-test. * p<0.05, ** p<0.01, *** p<0.001.

The MATLAB script was able to accurately segment DsRed+ and GFP+ colonies. GFP+ cells that had been pretreated with Paclitaxel before plating show significantly reduced median colony size, as compared to non-pretreated DsRed+ cells in co-culture (Fig 4A). This difference in colony sizes was also observed visually (S4A Fig).

We developed a method to quantitatively compare protein-expression between cell lines. The `Ki-67 index' statistic was defined as the fraction of cells in a colony expressing Ki-67. A colony with a Ki-67 index of 1.0 displays Ki-67 expression in all of its constituent cells The effects of Paclitaxel pretreatment on Ki-67 index are shown (Fig 4B and 4C). The cumulative frequency curve for RFP+ (non-pretreated) cells is relatively steep, indicating that majority of the colonies had >80% of cells expressing Ki-67. On the other hand, the GFP+ curve shows that a large number of low Ki-67 colonies were formed by Taxol pre-treated cells (Fig 4B). Approximately 20% of the GFP+ colonies were observed to be completely non-proliferative and did not express Ki-67 at all, as seen in the y-intercept of the GFP+ curve in Fig 4B. Images of DsRed+ and GFP+ colonies with Ki-67 staining are shown in S4 Fig.

Control wells, in which neither GFP+ nor DsRed+ were pretreated with paclitaxel show no differences in colony size distributions or Ki67 expression (S5 Fig). There was no significant difference between the proliferation rates of the untreated DsRed+ and GFP+ cell lines (S6A and S6B Fig). Finally, we validated colony segmentation by comparing our method to ImageJ algorithms. Colony images were binarized using Otsu’s algorithm, available in the ImageJ toolbox. The size distribution of the binarized objects was obtained by using the object counter available in ImageJ (S6C Fig). While the overall shape of the resulting colony size distribution is similar between methods, the probabilities for areas >5000 pixels are larger with ImageJ than with our method. We suspect that this is due to the fact that a binarization step alone, as used in ImageJ, is unable to distinguish between connected colonies.

### *In vitro* colony analysis of live samples

#### Image processing steps in live-cell mode

Next, we wished to determine whether we would get similar results using a single 6-well plate of cells imaged over time. We utilized our algorithm to analyze live, unstained samples. Twenty-four hours after mixing and plating DsRed+ and pre-treated GFP+ cells, we imaged each well to detect DsRed and GFP expression. The wells were re-imaged on days 2 and 4. This `live-cell' mode has the advantage that it requires fewer materials, no sample preparation and less imaging time. It is a dynamic assay and allows colonies to be imaged over time (S7 Fig). Since there is no nuclear staining or immunofluorescence staining, individual cells and protein expression cannot be identified.

The image processing scheme followed is similar to the fixed-cell mode in that we again employ a distance transform to identify `basins', which are used to segment colonies by applying the watershed algorithm. The difference between the fixed-cell and live-cell modes is in the channels to which the segmentation step is applied. In the fixed-cell mode, DAPI channel images displaying nuclear staining are used to identify individual colonies. In the live-cell mode, we directly segment colonies using images obtained from the RFP/GFP channel (i.e. displaying the native DsRed/ GFP fluorescence of the cell-lines under analysis).

**Fig 5:**
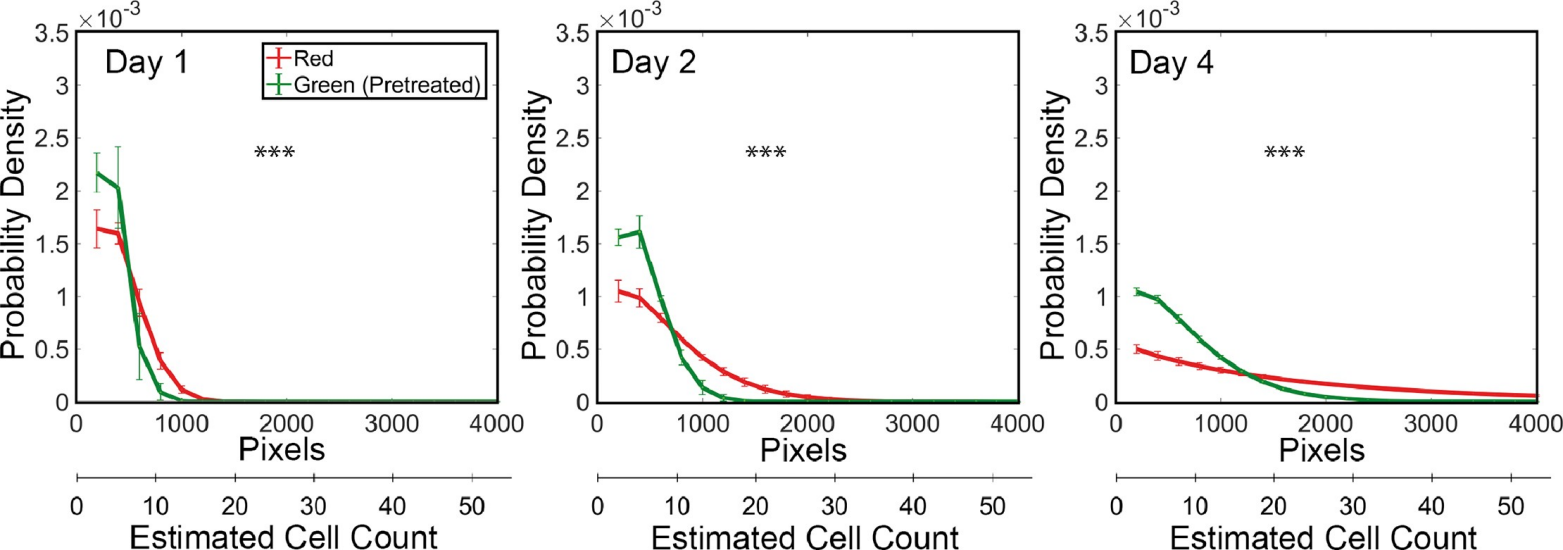
Live-cell assay results. A: The probability density functions of colony sizes as they change over the duration of the live cell experiments. Mean and standard deviations of probability distribution functions are shown, and represent biological replicates obtained from 3 wells of a 6 well-plate. Number of colonies > 150 per well. The X-axis shows colony area and estimated cell counts. Significance between mean ranks was determined using the Kruskal-Wallis test (*** p<0.001).

The GFP+ cell-line treated with paclitaxel prior to drug treatment produced smaller colonies than the untreated DsRed+ cells in co-culture (Fig 5, S7 Fig). Furthermore, it was possible to monitor colony growth and the associated change in colony size distributions over time. The live-cell mode produced similar colony size distributions and responses to paclitaxel pre-treatment over time, which makes it a useful approximation for the fixed-cell experiment (Fig 5).

Since the live-cell images do not have nuclear staining, it is not possible to segment individual cells as is done in the fixed-cell mode. Instead, in the live-cell mode, colony area is used to approximate colony size. However, colony areas and cell counts are well correlated and can be used to estimate the number of cells in a colony in live-cell experiments (S8 Fig).

### In vivo metastatic colony analysis and image processing

#### Introduction

A modified version of the colony segmentation algorithm described in the previous section was applied to analyze the size distribution of metastatic colonies across entire mice lung tissue sections. Slides were prepared from mouse lung tissue sections, which contained metastases constituted by DsRed+ and GFP+ MDA-MB-231 cells.

**Fig 6:**
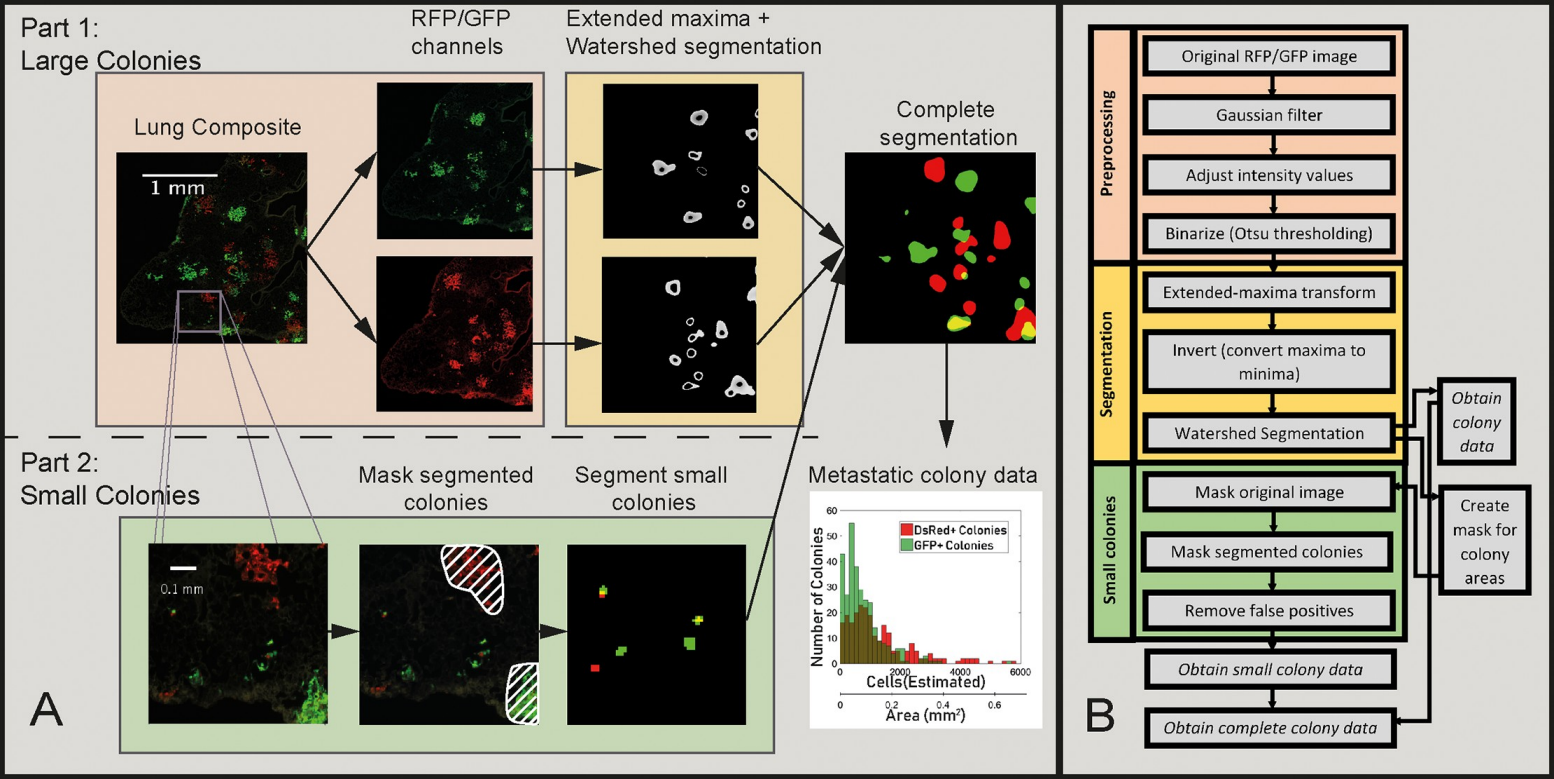
Image processing steps for *in vivo* images. A: Overall scheme for segmenting colonies in mice lung tissue sections. B: Flow diagram listing individual steps in the MATLAB script.

The slides were imaged, and the image processing scheme shown in Fig 6 was applied to obtain quantitative data. The algorithm is divided into two parts—Part 1 segments large metastatic colonies but not micrometastases (Fig 6A) which are eliminated during the blurring step. Once large colonies are segmented, they are masked and the remaining area is scanned for micrometastases, i.e. seeding events which are only a few cells large in size, shown in Part 2. Any such events that are within an arbitrary distance of 40 pixels from the large metastatic colonies are not counted, since we cannot know whether such a colony has been seeded from a primary tumor or represents cells migrating from the nearby metastatic colony in the organ. The DAPI channel is only used to identify lung regions from background and plays no role in the colony segmentation steps.

#### Extended-maximum transform

The extended-maximum transform is used to identify local maxima—which are regions of densely packed cells—in metastatic colonies, which serve as the foci (‘basins’/ ‘troughs’) for the subsequent watershed transformation step. A similar method has been discussed for other applications in existing literature [35]. The segmentation step is accurate even for regions of dense colonies as shown in S9 Fig. In such cases, a threshold-based binarization step alone is unable to distinguish between adjacent metastases. The extended-maximum transform is used instead of a distance transformation step in the *in vivo* algorithm. While distance transformation can be successfully employed in the *in vitro* segmentation algorithm, the greater variability inherent in lung tissue sections renders it unsuitable for the *in vivo* method.

#### *In vivo* experimental results: Lung colonies in tissue sections show no difference in size distributions

**Fig 7:**
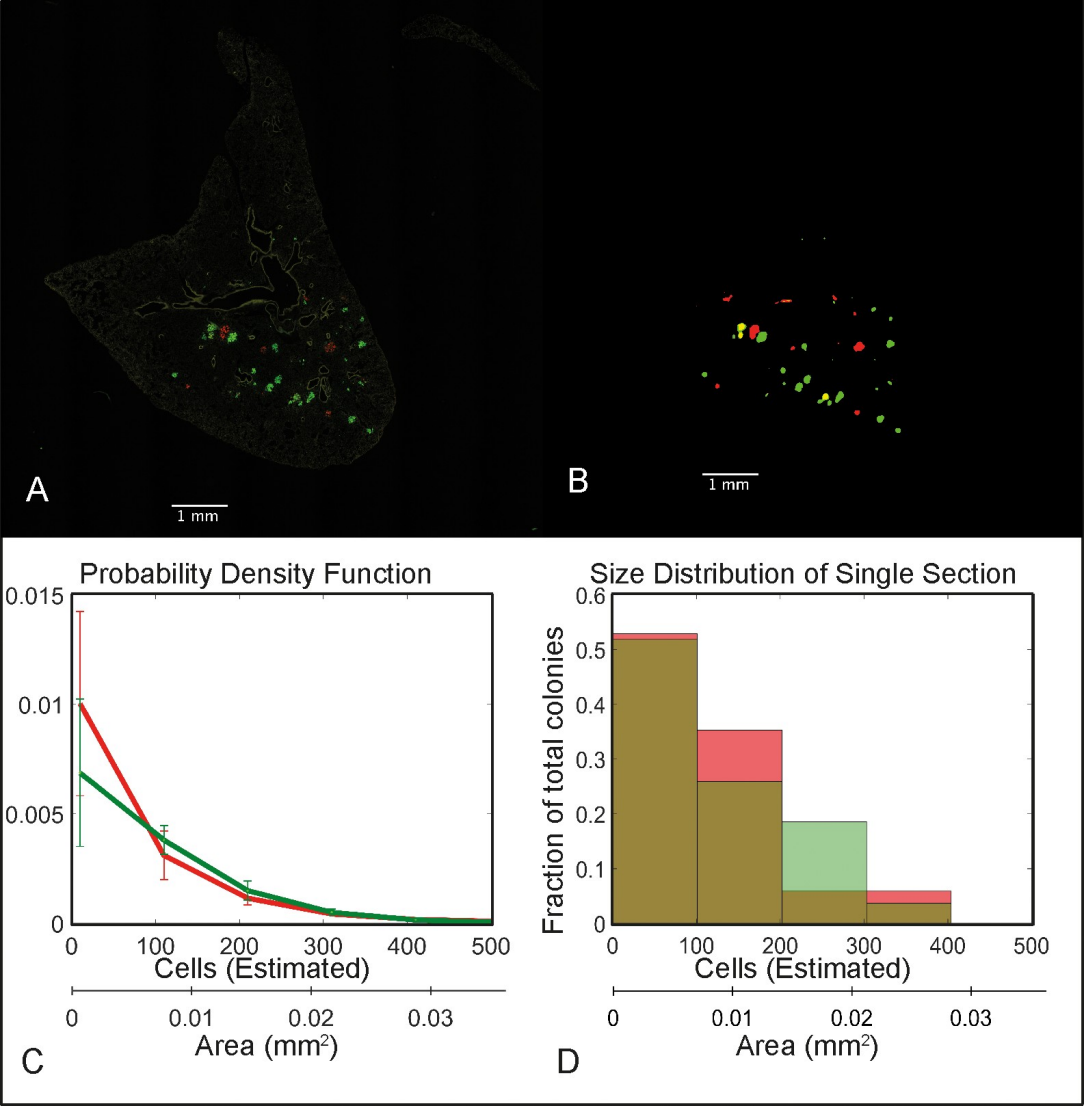
*In vivo* results. **A: Composite RFP/GFP image.** B: Metastatic colonies were identified. C: Colony size distribution of DsRed+ and GFP+ metastatic colonies in a single lung. D: Mean Probability Densities and standard deviations of GFP+ and DsRed+ colonies, from a set of three mice. Number of DsRed+ or GFP+ colonies in each mice section are greater than 10. No significant difference was obtained between the median colony sizes using the two-tailed Wilcoxon rank-sum test.

Metastatic colonies in lung sections of mice injected with DsRed+ and GFP+ MDA-MB-231 cells are shown (Fig 7A and 7B). The algorithm segments lung images (Fig 7B) and obtains size distributions for metastatic colonies (Fig 7C and 7D). No significant difference was obtained in the size distributions of the DsRed+ and GFP+ metastatic colonies in lungs.

**Fig 8:**
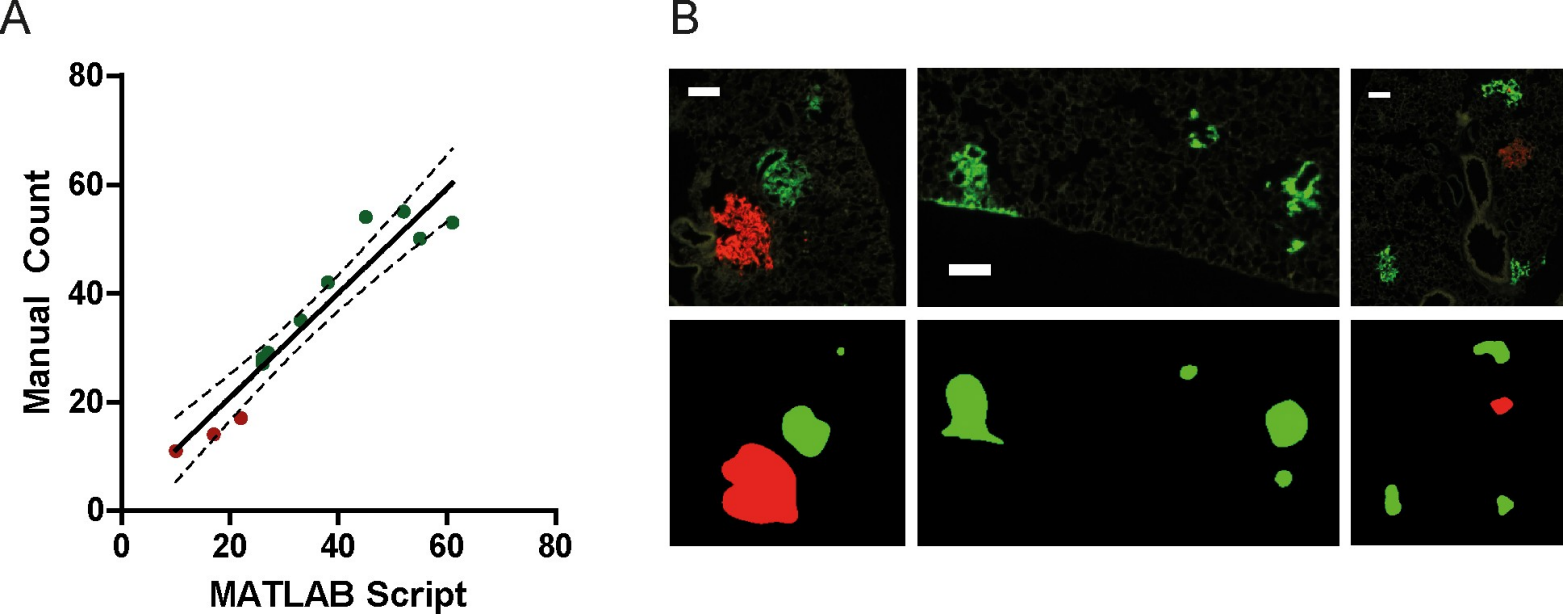
*In vivo* validation. A: Validation of metastatic counts. Each red/green point represents DsRed+/GFP+ metastatic colonies in one lung section respectively. B: Metastatic colonies and corresponding segmented areas.

The segmentation accuracy was validated by comparing the number of metastatic colonies obtained using our algorithm and by manual counting (Fig 8A), or by visual comparison of segmented areas (Fig 8B).

**Fig 9:**
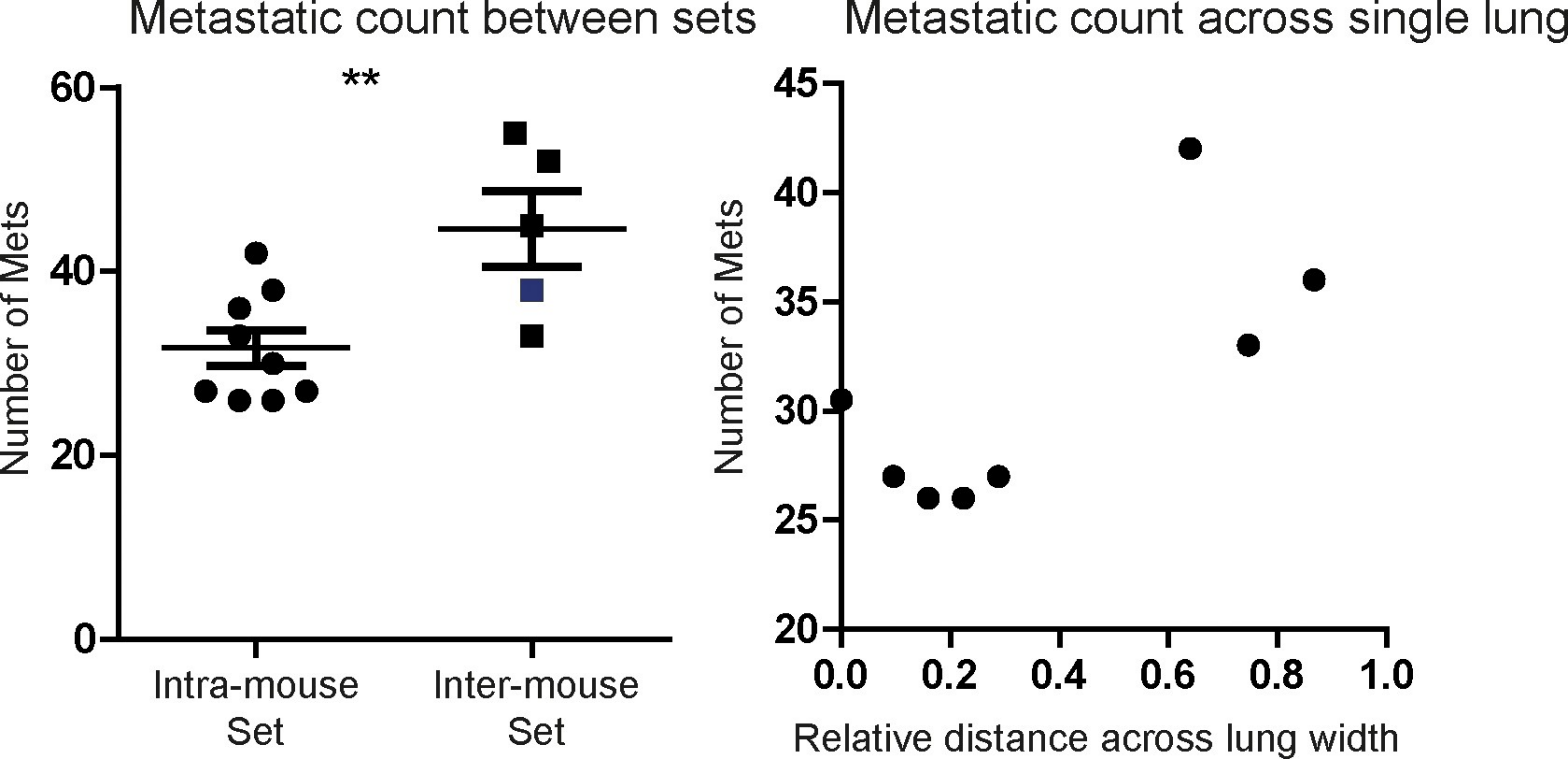
Intra-mouse vs mouse-to-mouse variation. A: Metastatic counts in intra-mouse (N = 8) and inter-mouse (N = 5) lung tissue sections. Blue data point represents mouse used for generating intra-mouse lung sections. B: Metastatic counts from the intra-mouse set, arranged according to distance across the lung width.

Furthermore, we compared experimental mouse-to-mouse variation, and variation within a single lung across 8 serial sections (Fig 9). The set of intra-mouse lung sections consisted of eight GFP-channel images, serially sectioned at 8 different points along a 1 mm thickness. The inter-mouse set consisted of single images obtained from 8 different mice lungs. The highlighted data point represents the mouse from which the intra-mouse lung sections were generated. We observe that the mouse-to-mouse variation is greater than across the serial sections in the same mouse (Fig 9A).

## Discussion

Quantitative data on metastatic colony sizes and their spatial distribution in the organ allows the experimenter to view metastatic seeding events and analyze growth rates *in vivo* [36]. Metastatic seeding and growth at the metastatic site are required for developing computational models for cancer metastasis. However, existing methods of analyzing metastasis in mice are unable to capture this information. The MATLAB tool described in this work segments metastatic colonies in mice lung tissue sections and obtains colony size distributions for fluorescent cell lines. The *in vitro* scheme of this tool can be used to segment fluorescent cell colonies in well plates and quantify expression of immunostained proteins. This represents an improvement over the clonogenic assay, which has seen wide use over the years in cancer research [31,37].

While currently available software such as ImageJ allows plugins that use similar morphological operators, we believe this script offers an easy way to control and change various parameters of the algorithm to suit the needs of a given experimental set. For example, within the MATLAB script and its functions changing binarization threshold, the limit of the h-minimum transform, etc. can be quickly and easily carried out and the results visualized.

Watershed-based segmentation methods have been used to identify objects in digital pathology [38–40]. Our work represents another application of watershed segmentation for identifying objects, namely cell colonies in lung tissue sections and well plates. In our work both, the *in vitro* and *in vivo* segmentation schemes make use of the watershed algorithm in identifying colonies. The segmentation algorithm we employ is fast, accurate and reliable for even densely seeded samples. It is suitable for quantitatively analyzing fluorescently tagged cell lines which are becoming increasingly popular in cancer research. Given the high resolution and brightness of fluorescent markers, imaging at a magnification of 4x is sufficient for effective analysis. This obviates any need for specialized whole slide imaging platforms and software requirements for image acquisition or large-image handling[41].

As a next step, the *in vitro* assay could be modified to also identify cell-cycle phases (G1, G2, S) of cells. Successful image analysis based cell-cycle quantification has been demonstrated in literature [42] and could add an interesting dimension to the colony-formation assay we describe. The *in vivo* assay could be expanded to include measurements of immunostained proteins-of-interest, such as Ki-67.

Furthermore, improvements to segmentation accuracy and sensitivity of the algorithm, especially for different metastatic organs, such as brain, liver, lymph nodes would be useful. The liver suffers from high autofluorescence leading to lower segmentation accuracy. Machine-learning techniques are a promising avenue for improving segmentation, since we expect to produce a large number of fluorescence-microscopy images which may be used as training sets. Extending this methodology to H&E stained sections would widen the scope of this work significantly. While some efforts have been taken in this direction, accurate segmentation in H&E stained images remains challenging [8,39].

## Supporting information

S1 Fig Lentiviral vectors. A: Vector 1, leading to expression of DsRed. B: Vectors 1 and 2, leading to cleavage of vector 1, expression of GFP and degradation of DsRed.
(TIF)

S2 Fig *In vitro* image processing steps. A: Colony image in the GFP channel. B: Preprocessing steps applied to A. C: Single Colony DAPI channel image. D: Distance transform applied to C.
(TIF)

S3 Fig *In vitro* segmentation examples. A-C: Successful segmentation of connected colonies (A) via distance transform (B) and subsequent watershed segmentation (C). D-F: Fused colonies segmented incorrectly. Scalebars = 1 mm.
(TIF)

S4 Fig Segmentation of dense colonies *in vivo*. A: DsRed+ and GFP+ colonies, GFP and RFP channel composite B: DAPI and CY5 channel composite, showing higher fraction of nuclei positive for Ki-67 in DsRed+ colonies. Scale bars = 450 microns.
(TIF)

S5 Fig *In vitro* negative control results. A: Colony size distributions show no significant differences between DsRed+ and GFP+ cell lines. Significance was tested using the Wilcoxon rank-sum test. B: Identical cumulative frequency distribution of Ki-67 Index. C. Ki-67 expression levels show no significant difference between DsRed+ and GFP+ cell lines. Significance was tested using unpaired t-test.
(TIF)

S6 Fig Native proliferation rates and method validation. A: Non-pretreated DsRed+ and GFP+ cells show no significant difference in confluence fold change. For cells pretreated with Paclitaxel, confluence values are reduced (*** p<0.001) B: Cell proliferation rates tested using hemocytometer counts over time. No significant differences between proliferation rates of DsRed+ (Red) and GFP+ (Green) cells. C: Comparing segmentation by MATLAB tool to binarizing and object counting in ImageJ.
(TIF)

S7 Fig In vitro live cell image processing steps. A,B: RFP/GFP channel images after preprocessing. C: Combined results of segmenting RFP and GFP channels by distance transform and a subsequent watershed algorithm. D: Colony level metrics obtained from segmented images. E. Colony size distributions obtained sorted according to cell line (Significance tested by Kruskal-Wallis test, *** p<0.001). Scale bars = 1 mm.
(TIF)

S8 Fig Correlation between colony area and cell counts for MCF7 cells *in vitro*. Using the *in vitro* fixed-cell assay, we obtain both areas and cell counts for each colony. This information can be used for a given cell line to estimate colony cell counts for subsequent live-cell experiments.
(TIF)

S9 Fig Segmentation of dense colonies *in vivo*. A: Lung GFP channel image of metastatic colonies. B: Results obtained by MATLAB tool. C. Binarization step in ImageJ does not segment individual colonies. Scalebar = 1 mm.
(TIF)

S10 Fig Example of reducing image size. To cover the majority of a single well in a 6-well plate required imaging 180 images (4X objective) per channel that were stitched. Each original image consists of 13346 X12300 pixels at 1.346 micron per pixel (left) which was reduced to 6673 X 6150 pixels at 2.692 micron per pixel (right) as shown.
(PDF)
