# Supplementary figures and images for: A software tool for the quantification of metastatic colony growth dynamics and size distributions in vitro and in vivo
Source: PLoS One. 2018 Dec 27;13(12):e0209591. doi: 10.1371/journal.pone.0209591 (PMC6307751; doi:10.1371/journal.pone.0209591)

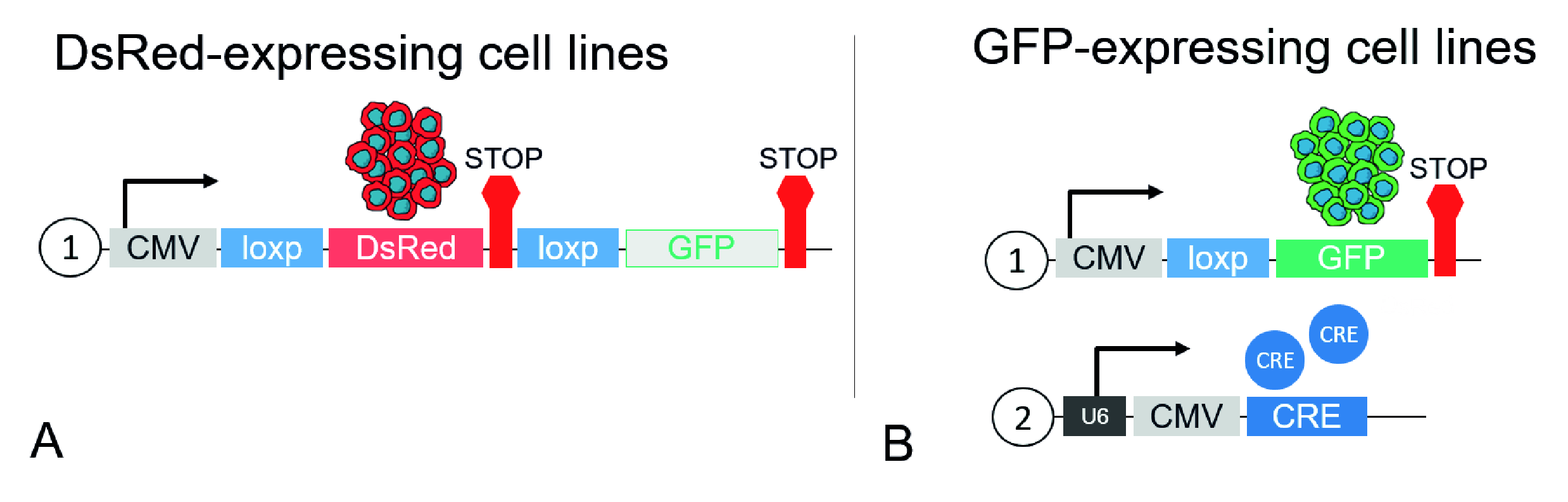

Supplement: S1 Fig — A: Vector 1, leading to expression of DsRed. B: Vectors 1 and 2, leading to cleavage of vector 1, expression of GFP and degradation of DsRed. (TIF) [file pone.0209591.s001.tif]

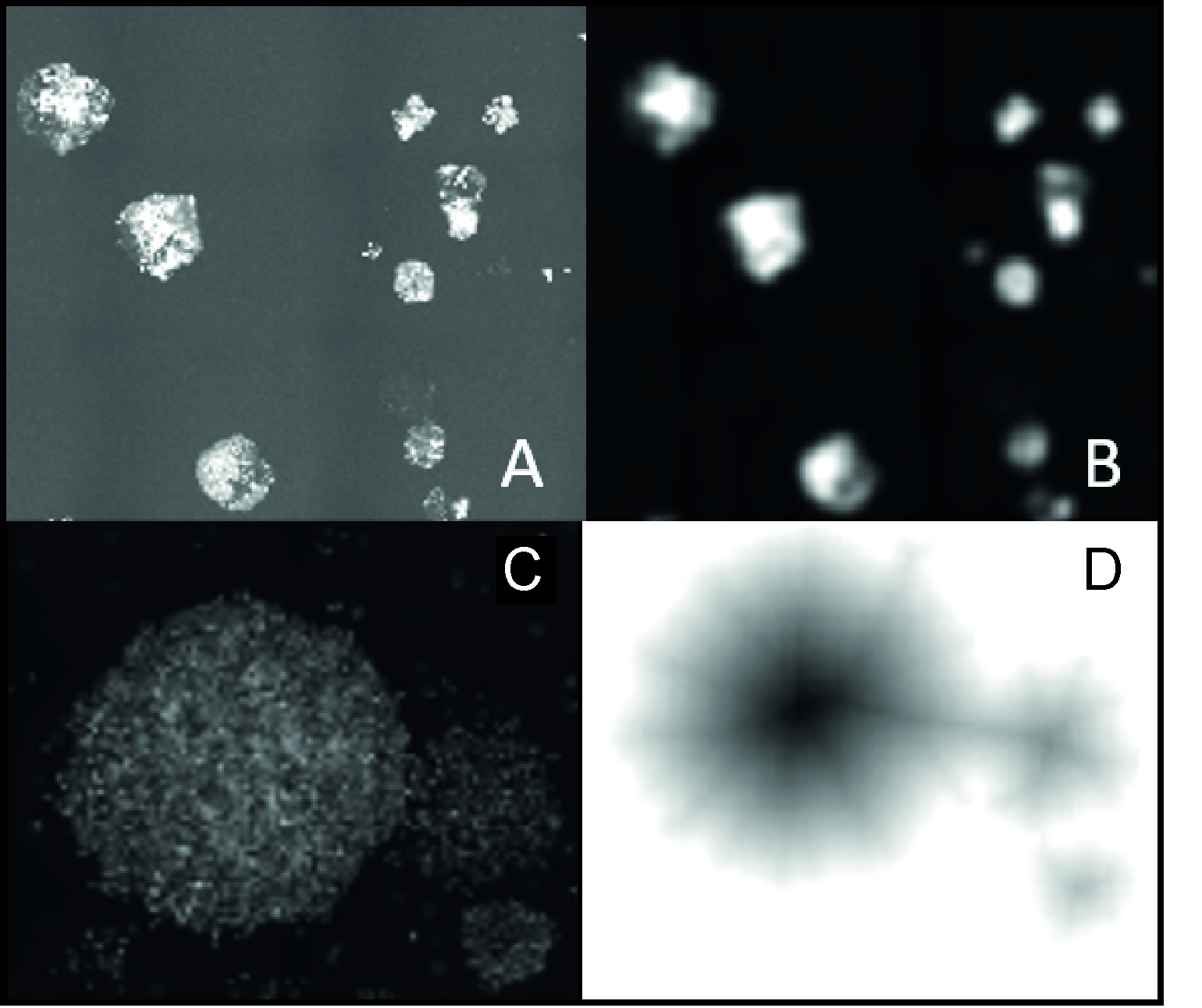

Supplement: S2 Fig — A: Colony image in the GFP channel. B: Preprocessing steps applied to A. C: Single Colony DAPI channel image. D: Distance transform applied to C. (TIF) [file pone.0209591.s002.tif]

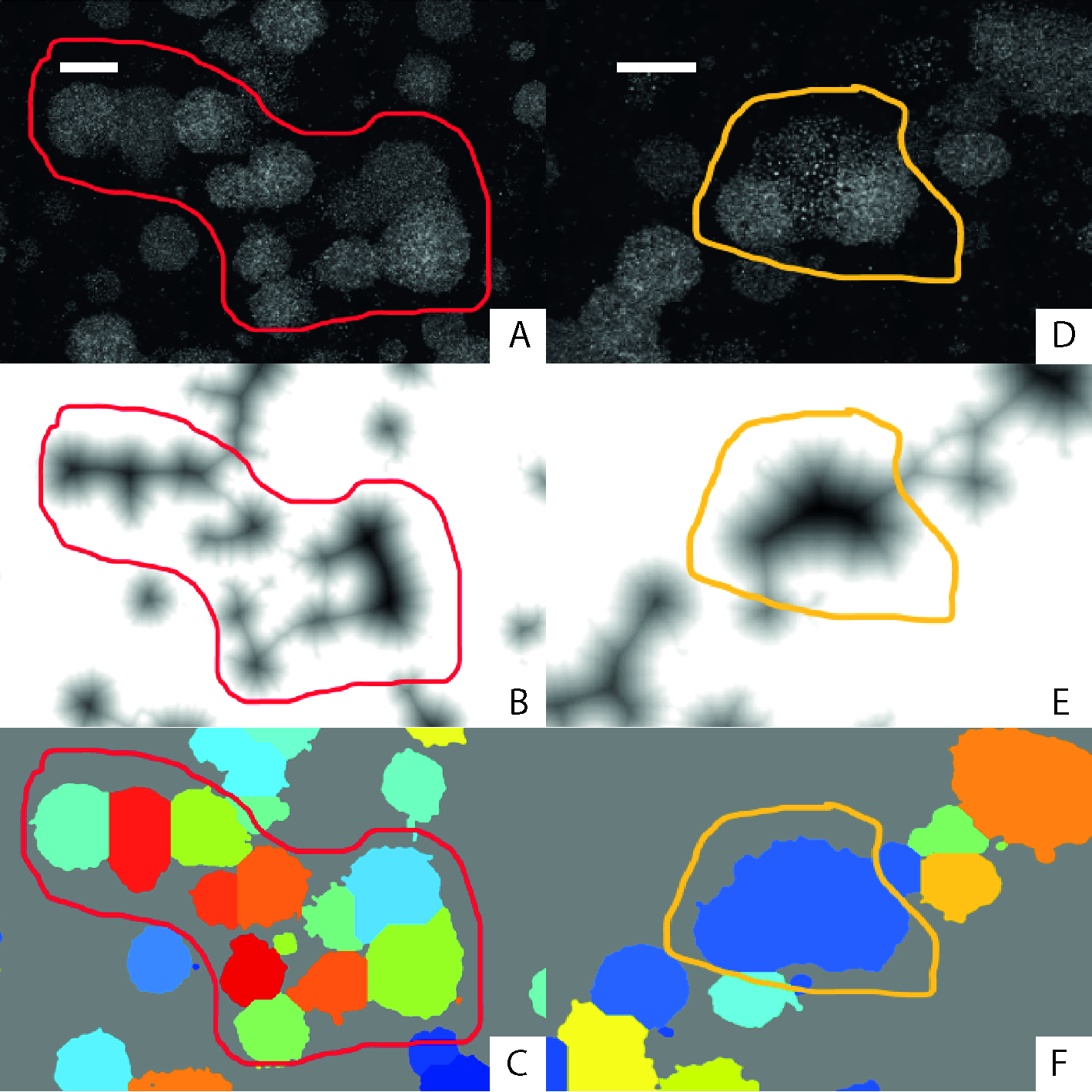

Supplement: S3 Fig — A-C: Successful segmentation of connected colonies (A) via distance transform (B) and subsequent watershed segmentation (C). D-F: Fused colonies segmented incorrectly. Scalebars = 1 mm. (TIF) [file pone.0209591.s003.tif]

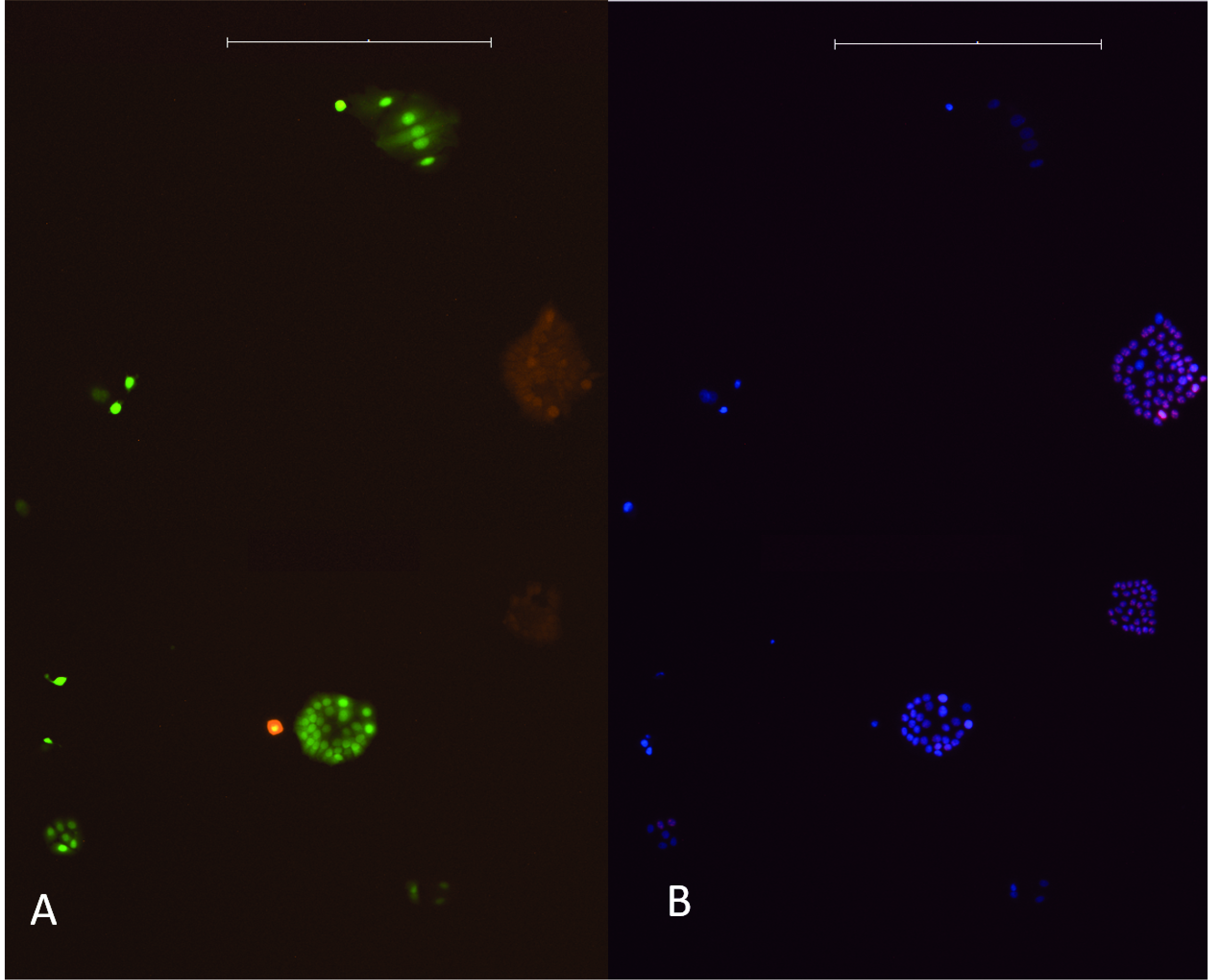

Supplement: S4 Fig — A: DsRed+ and GFP+ colonies, GFP and RFP channel composite B: DAPI and CY5 channel composite, showing higher fraction of nuclei positive for Ki-67 in DsRed+ colonies. Scale bars = 450 microns. (TIF) [file pone.0209591.s004.tif]

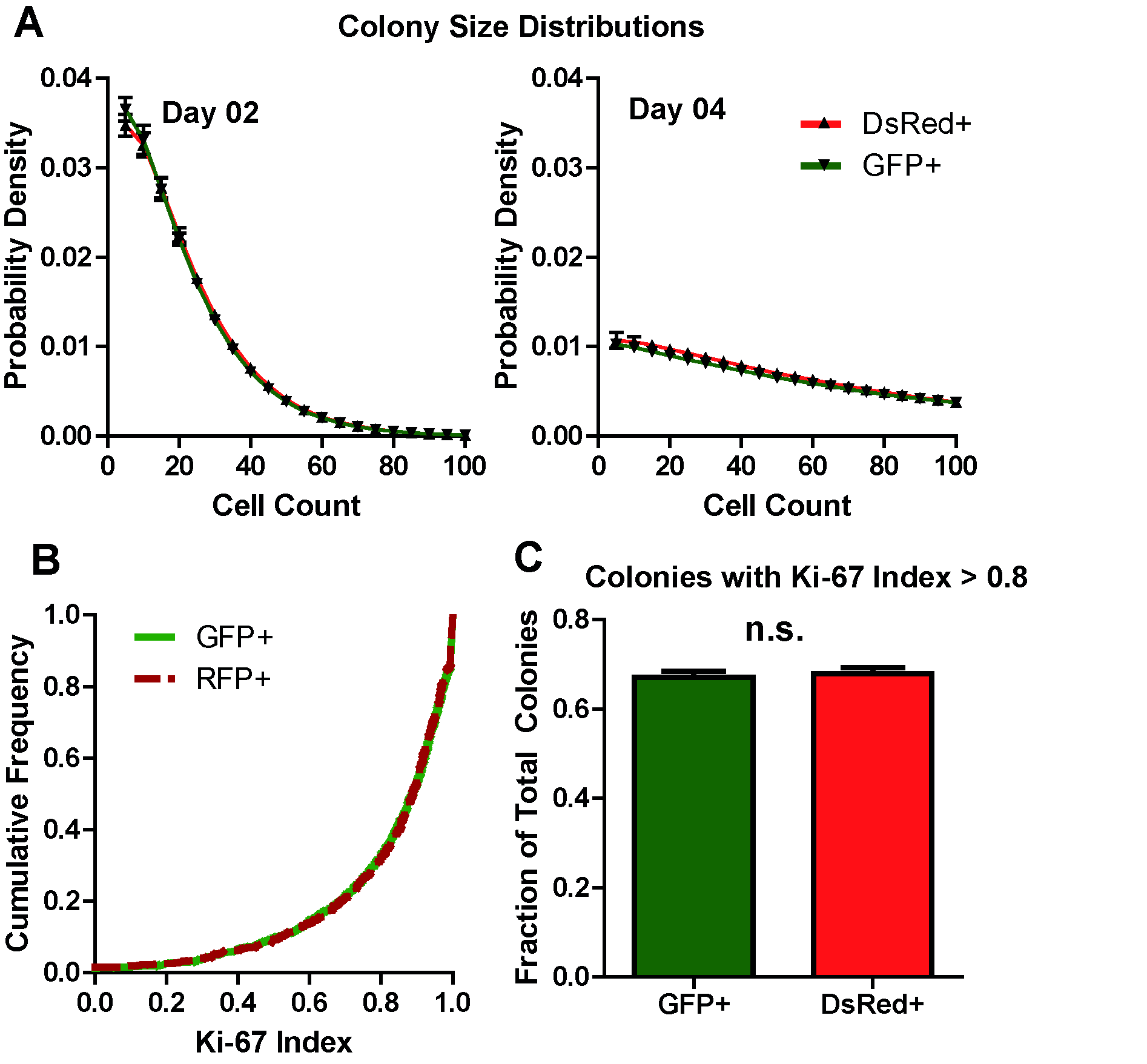

Supplement: S5 Fig — A: Colony size distributions show no significant differences between DsRed+ and GFP+ cell lines. Significance was tested using the Wilcoxon rank-sum test. B: Identical cumulative frequency distribution of Ki-67 Index. C. Ki-67 expression levels show no significant difference between DsRed+ and GFP+ cell lines. Significance was tested using unpaired t-test. (TIF) [file pone.0209591.s005.tif]

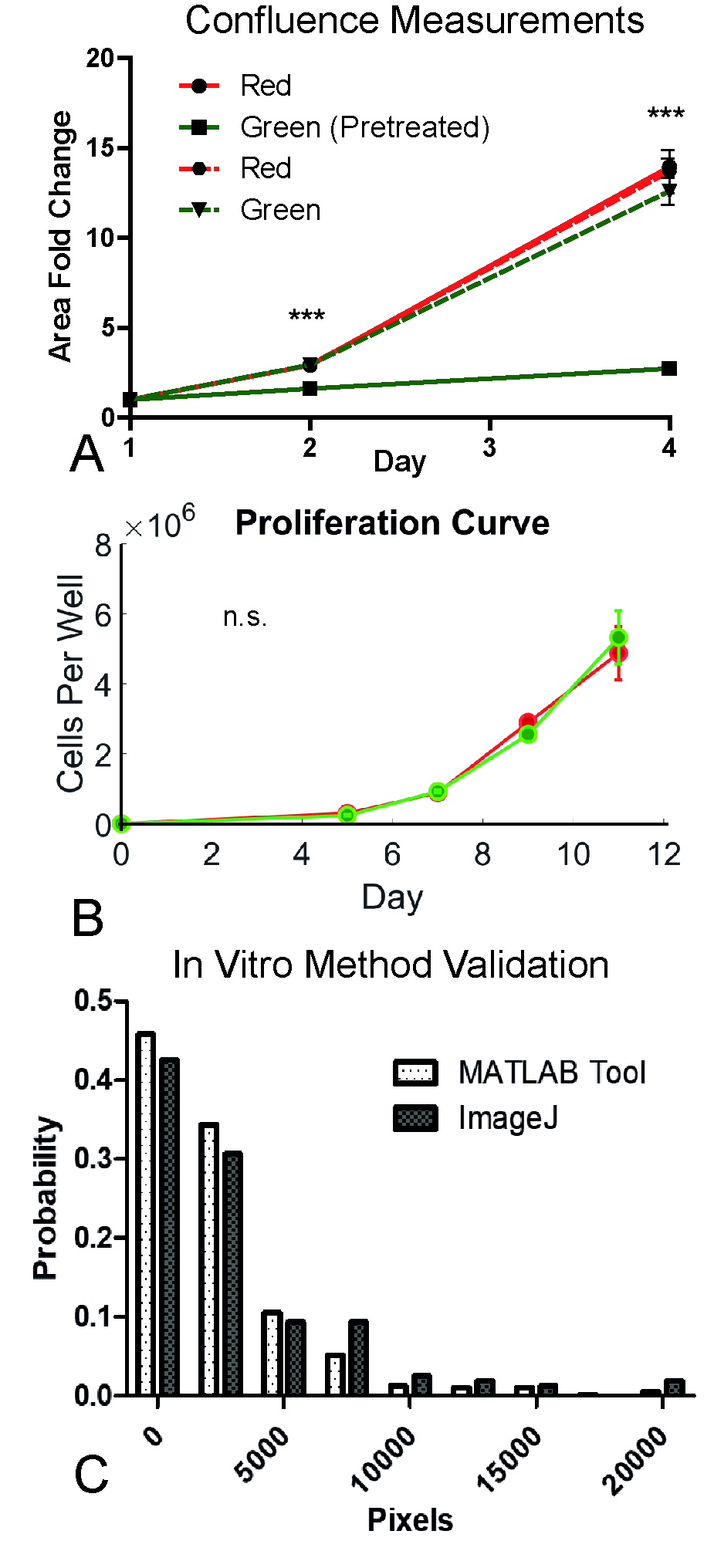

Supplement: S6 Fig — A: Non-pretreated DsRed+ and GFP+ cells show no significant difference in confluence fold change. For cells pretreated with Paclitaxel, confluence values are reduced (*** p<0.001) B: Cell proliferation rates tested using hemocytometer counts over time. No significant differences between proliferation rates of DsRed+ (Red) and GFP+ (Green) cells. C: Comparing segmentation by MATLAB tool to binarizing and object counting in ImageJ. (TIF) [file pone.0209591.s006.tif]

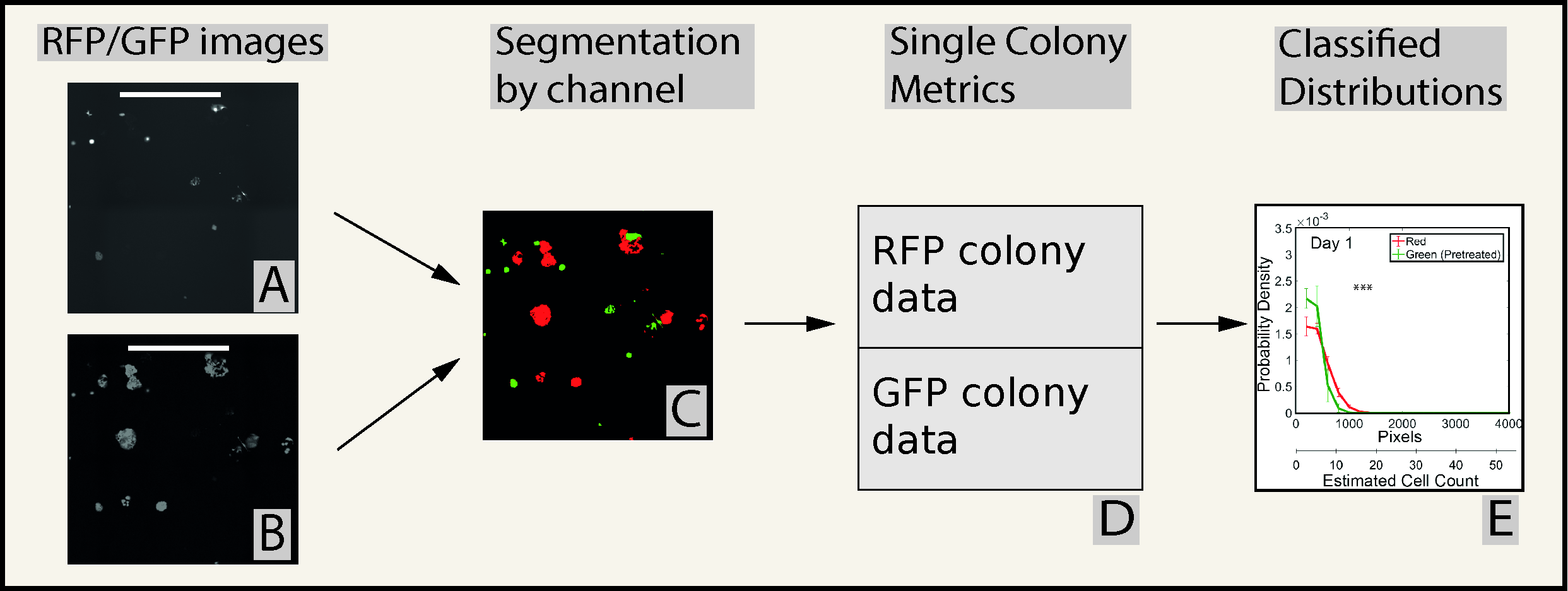

Supplement: S7 Fig — A,B: RFP/GFP channel images after preprocessing. C: Combined results of segmenting RFP and GFP channels by distance transform and a subsequent watershed algorithm. D: Colony level metrics obtained from segmented images. E. Colony size distributions obtained sorted according to cell line (Significance tested by Kruskal-Wallis test, *** p<0.001). Scale bars = 1 mm. (TIF) [file pone.0209591.s007.tif]

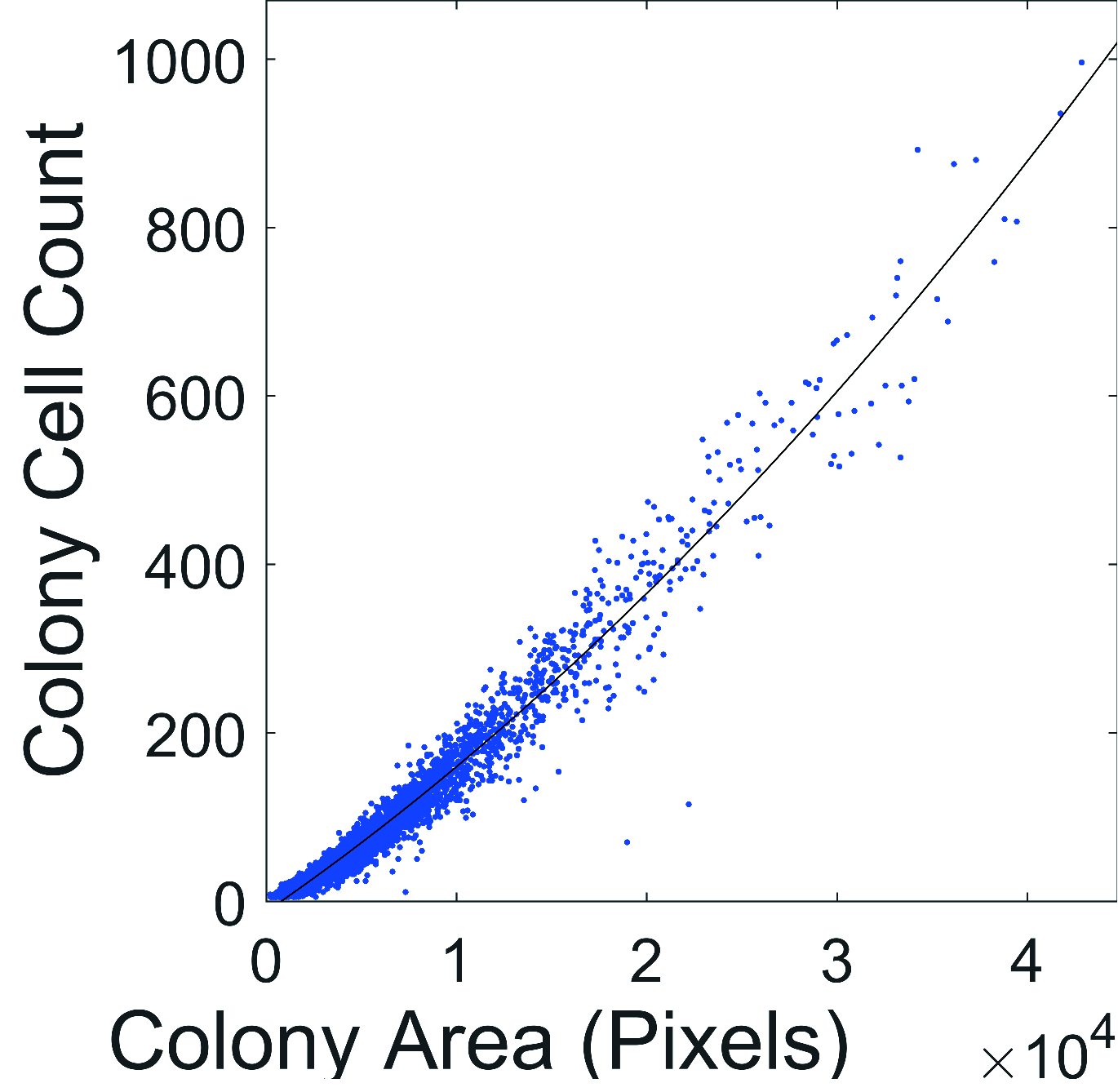

Supplement: S8 Fig — Using the in vitro fixed-cell assay, we obtain both areas and cell counts for each colony. This information can be used for a given cell line to estimate colony cell counts for subsequent live-cell experiments. (TIF) [file pone.0209591.s008.tif]

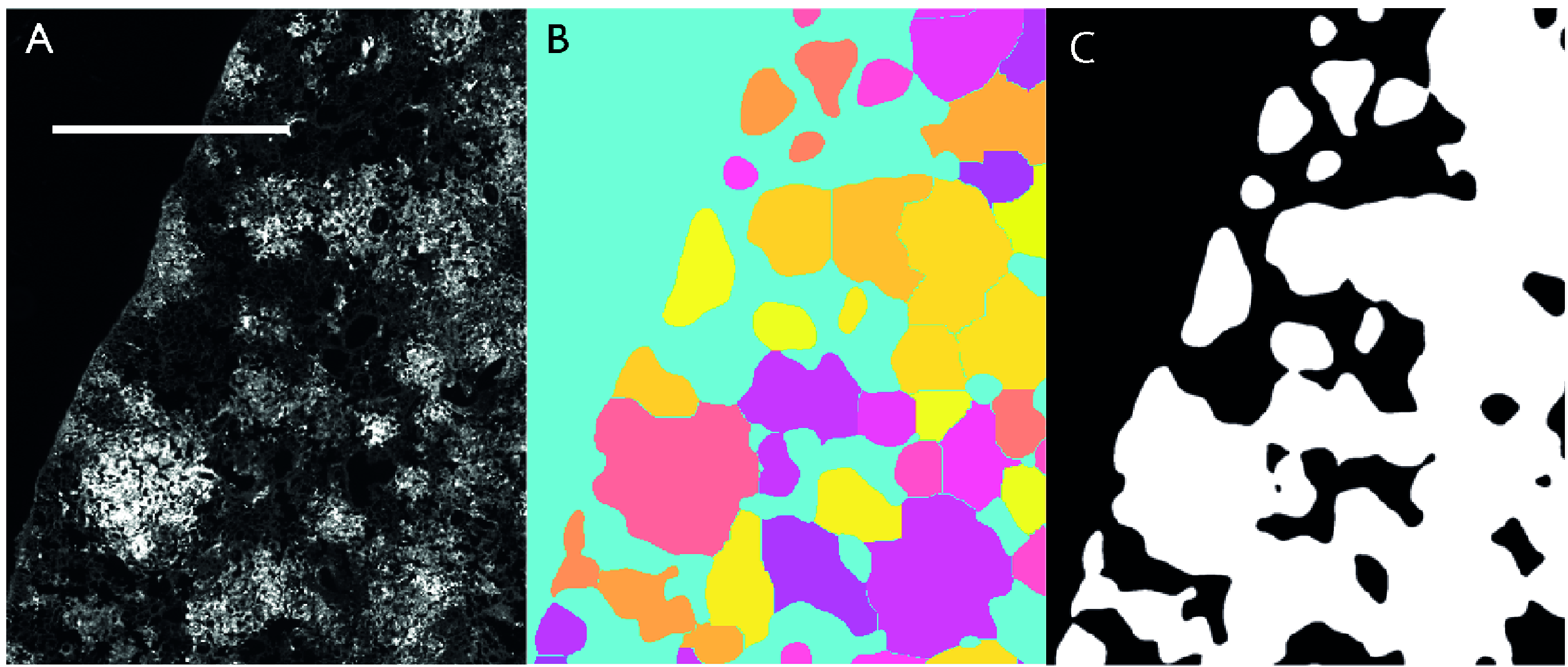

Supplement: S9 Fig — A: Lung GFP channel image of metastatic colonies. B: Results obtained by MATLAB tool. C. Binarization step in ImageJ does not segment individual colonies. Scalebar = 1 mm. (TIF) [file pone.0209591.s009.tif]
