# Supplementary material for: A software tool for the quantification of metastatic colony growth dynamics and size distributions in vitro and in vivo
Source: PLoS One. 2018 Dec 27;13(12):e0209591. doi: 10.1371/journal.pone.0209591 (PMC6307751; doi:10.1371/journal.pone.0209591)
Supplement: S10 Fig — To cover the majority of a single well in a 6-well plate required imaging 180 images (4X objective) per channel that were stitched. Each original image consists of 13346 X12300 pixels at 1.346 micron per pixel (left) which was reduced to 6673 X 6150 pixels at 2.692 micron per pixel (right) as shown. (PDF) [file pone.0209591.s010.pdf]

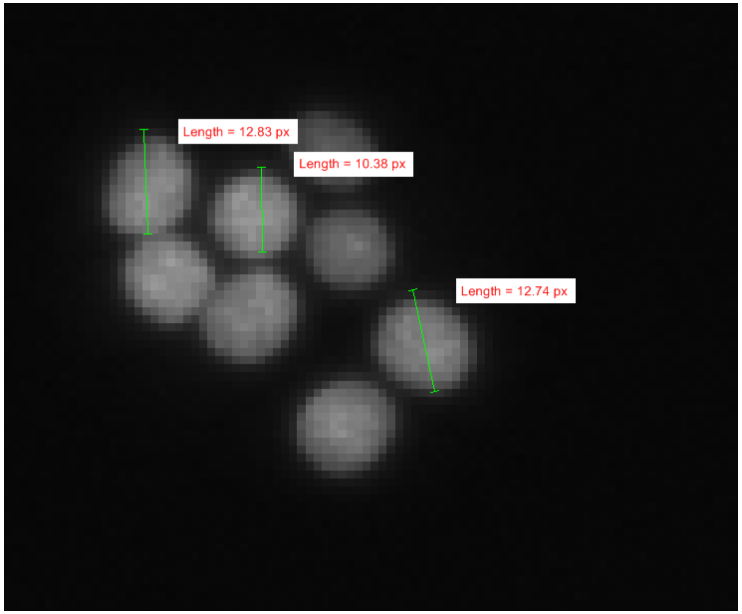

Original Image
1.346 um/px
13346 x 12300 px total

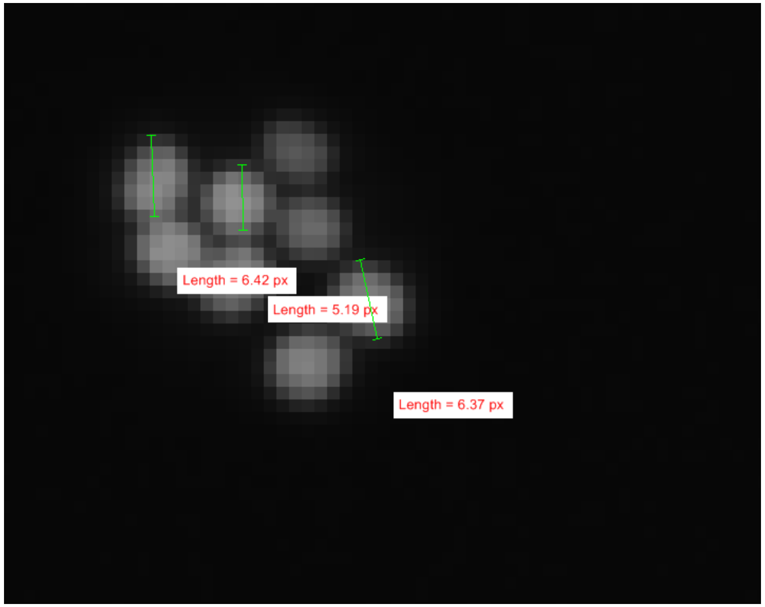

Reduced image used as input to the script
2.692 um/px
Total 6673 x 6150 px
